# Effects of glucagon-like peptide-1 on systemic hemodynamics, kidney function, and intrarenal oxygenation in sheep with sepsis-associated acute kidney injury

**DOI:** 10.1038/s41598-025-33109-0

**Published:** 2025-12-24

**Authors:** Abraham H. Hulst, Connie P. C. Ow, Clive N. May, Sally G. Hood, Mark P. Plummer, Jeroen Hermanides, Daniël H. van Raalte, Adam M. Deane, Rinaldo Bellomo, Yugeesh R. Lankadeva

**Affiliations:** 1 Preclinical Critical Care Unit, Florey Institute of Neuroscience and Mental Health, University of Melbourne, Melbourne, VIC Australia; 2 Department of Anesthesiology, Amsterdam UMC, University of Amsterdam, Meibergdreef 9, 1105 AZ Amsterdam, The Netherlands; 3 Department of Intensive Care, Royal Adelaide Hospital, Adelaide, Australia; 4 Department of Internal Medicine, Amsterdam UMC, University of Amsterdam, Amsterdam, Netherlands; 5 Department of Intensive Care, Royal Melbourne Hospital, Melbourne, VIC Australia; 6 Department of Critical Care, Melbourne Medical School, The University of Melbourne, Melbourne, VIC Australia; 7 Australian and New Zealand Intensive Care Research Centre, Monash University, Melbourne, VIC Australia; 8 Department of Intensive Care, Austin Hospital, Melbourne, VIC Australia; 9 Department of Anesthesia, Austin Hospital, Melbourne, VIC Australia

**Keywords:** Glucagon-like peptide-1, Sepsis, Acute kidney injury, Renal medullary oxygenation, Sheep, Diseases, Medical research, Nephrology, Physiology

## Abstract

Glucagon-like peptide-1 receptor agonists (GLP-1 RAs) reduce chronic kidney disease progression in people with type 2 diabetes mellitus. Sepsis is the leading cause of acute kidney injury (AKI). This study investigated whether GLP-1 is renoprotective in an ovine model of gram-negative septic AKI. Sixteen healthy merino ewes were surgically instrumented to measure mean arterial pressure, cardiac output, renal blood flow, renal cortical and medullary perfusion and oxygenation, and renal function. After a 5-day recovery period, sepsis was induced via continuous intravenous infusion of live *Escherichia coli* for 30 h. After 24 h, the sheep were randomized to receive an intravenous infusion of 3.6 pmol/kg/min GLP-1 (*n* = 8) or a fluid-matched vehicle (*n* = 8) for 6 h. After 24 h of sepsis, 7/8 sheep in each group developed AKI. GLP-1 treatment increased renal blood flow compared to placebo + 13 vs. − 3.4 ml/min difference (95% CI) = 16 (− 7–40) *P* = 0.0054), and maintained renal cortical oxygenation + 2.5 mmHg vs. = − 7.2 mmHg, difference (95% CI) = 9.7 (5.2–14.4) *P* < 0.001. GLP-1 maintained renal medullary perfusion + 1.7 vs. vehicle − 213 perfusion units, difference (95% CI) = 214 (− 21–450) *P* = 0.07. However, GLP-1 did not significantly improve the primary endpoint of renal medullary oxygenation − 1.6 vs. − 11.5, difference (95% CI) = 9.9 (− 6.8–26.7) *P* = 0.21. In an ovine model of gram-negative sepsis-associated AKI, GLP-1 infusion supported global renal perfusion, renal oxygen delivery, and cortical oxygenation but failed to improve renal medullary oxygenation and kidney function.

**Supplementary Information:**

The online version contains supplementary material available at 10.1038/s41598-025-33109-0.

## Introduction

Sepsis is the leading cause of acute kidney injury (AKI) in intensive care units (ICUs)^1^. Compared with either disease alone, sepsis-associated AKI (SA-AKI) has a worse prognosis^1,2^. SA-AKI is associated with a prolonged length of stay in the ICU, increased mortality, increased likelihood of developing chronic kidney disease, and reduced quality of life^3–6^. Apart from treatment of the underlying infection and source control, general supportive measures for managing patients with sepsis-associated AKI include preservation of tissue oxygenation, correction of hypovolemia and hypotension, and avoidance of nephrotoxins. However, specific targeted therapies to prevent or treat AKI in the context of sepsis are lacking^7^.

Treatment with glucagon-like peptide-1 receptor agonists (GLP-1 RAs) is an established therapy for patients with type 2 diabetes that improves glucose control, induces weight loss, and reduces major adverse cardiovascular events. In these studies, GLP-1 RAs also reduced the incidence of important kidney events and kidney failure^8^. In addition, a mechanistic imaging study revealed that GLP-1 increases the perfusion and oxygenation of healthy human kidneys^9^. The effects of GLP-1 have been evaluated clinically in patients admitted to the ICU for their effects on blood glucose and gastric emptying^10^. To date, the effects of GLP-1 on renal macro- and microcirculation, kidney function and renal histopathology have not been studied in the context of sepsis-associated AKI.

During hyperdynamic septic AKI, there is an uncoupling of the renal macrocirculation and microcirculation. Despite preserved renal blood flow and oxygen delivery, localized tissue ischemia and hypoxia can occur, with the renal medulla particularly susceptible to developing hypoxia during the early stages of sepsis^11^. The onset of renal medullary hypoxia in sepsis is an early marker of the development of AKI^11,12^. Reducing renal medullary hypoxia can prevent AKI in experimental gram-negative sepsis^13^. Given the critical role of renal medullary hypoxia in the pathogenesis of sepsis-associated AKI, and evidence that reducing medullary hypoxia can prevent AKI in experimental sepsis, we selected renal medullary tissue oxygenation as our primary endpoint.

We aimed to investigate whether GLP-1 infusion could alleviate renal tissue hypoperfusion and hypoxia in an ovine model of live gram-negative SA-AKI. We hypothesized that GLP-1 would improve renal cortical and medullary tissue perfusion, oxygenation and kidney function in sheep with established septic AKI.

## Materials and methods

### Animals

Sixteen healthy female merino ewes (35–45 kg body weight) were housed in individual metabolic cages with free access to water and 800 g/day oaten chaff. The animals were procured from a farm in Central Victoria, Australia, and delivered into our facility at the Florey Institute for acclimatization before any experimentation. The Animal Ethics Committee of the Florey Institute of Neuroscience and Mental Health (Ethics identification number: 21-030-FINMH) approved these experiments under the guidelines of the National Health and Medical Research Council of Australia. All methods and procedures were performed according to these guidelines and regulations. This report was written in accordance with the ARRIVE 2.0 guidelines^14^.

The ewes underwent two aseptic surgical procedures under general anesthesia, described in detail previously^12,15–23^ and summarized below. Anesthesia was induced with intravenous sodium thiopentone (15 mg/kg, Jurox Pty Ltd, Rutherford, NSW, Australia) and maintained with isoflurane (2.0–2.5% v/v oxygen/air/isoflurane) following intubation. Prior to incision, the sheep was given 900 mg of the antibiotic procaine penicillin (Ilium Propercillin, Troy Laboratories, Glendenning, NSW, Australia) and 1 mg/kg of the analgesic flunixin meglumine (Ilium Flunixil, Troy Laboratories). First, the left carotid artery was exteriorized into a skin fold to form a carotid arterial loop for subsequent access to arterial cannulation^17,21^. A 20-mm transit-time flow probe (Transonic Systems, Ithaca, NY) for cardiac output (CO) measurement was placed around the pulmonary artery^17^. Three weeks later, the carotid artery was cannulated and connected to a pressure transducer for measurement of systolic, diastolic, and mean arterial pressure (SBP, DBP, and MAP), heart rate and blood sampling^21^. Three catheters were inserted into the right jugular vein: one for delivery of treatment, one for administering *E. coli*, and one for fluid resuscitation and vasopressors, as needed. The arterial and venous catheters were continuously infused with heparinized saline (10 U heparin/mL at 3 mL/h) to maintain patency. During the second surgical procedure, a 4-mm transit-time flow probe (Transonic Systems) was placed around the left renal artery to measure renal blood flow (RBF)^17^, and the left renal vein was cannulated for blood sampling. Two fiber-optic probes (Oxford Optronix, Abingdon, United Kingdom) were inserted into the renal cortex and medulla to measure renal cortical and medullary oxygenation (PrcO_2_, RrmO_2_) and perfusion (RCP, RMP)^15,22^. A Foley catheter was inserted into the bladder with a fiber-optic probe inserted into the tip to measure partial urinary oxygen pressure (PuO_2_)^12,15^. For both surgical procedures, the animals were injected with intramuscular antibiotics (900 mg procaine penicillin, Ilium Propen, Troy Laboratories, Smithfield, NSW, Australia) and analgesics (Flunixin meglumine, 1 mg/kg; Troy Laboratories or Mavlab) at the start of surgery and 24 and 48 h after surgery^15,18,23^. The animals were allowed at least five days after the second surgical procedure to recover prior to experimental intervention.

**Fig. 1:**
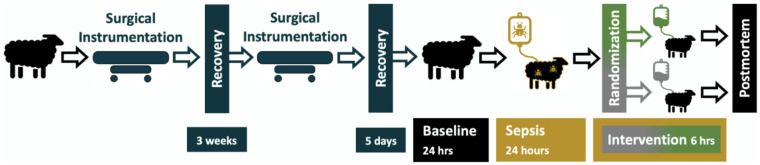
Study overview and experimental timeline.

### Experimental protocol

A schematic representation of the experimental protocol and data collection time points is depicted in Fig. 1. Following a 24-h baseline period, sepsis was induced in nonanesthetized sheep with an intravenous dose of live *E. coli* (2.8 × 10^9^ colony-forming units [CFUs] over 30 min) as a bolus, followed by a continuous infusion (1.26 × 10^9^ CFU/hr for the rest of the experiment). At 23.5 h of sepsis, fluid bolus therapy with Hartmann’s solution (Baxter Australia, 30 mL/kg over 30 min) was administered^23^. At 24 h after sepsis, the animals were randomized via online software built into electronic data capture software (Castor EDC, Castor B.V., Amsterdam, Netherlands).

The animals in the intervention group received an IV GLP-1 infusion of 3.6 pmol/kg/min for six hours (Bachem AG., Bubendorf, Switzerland) dissolved in 10 ml of 20% Albumin (CSL Behring, Broadmeadows, VIC, Australia). The total volume of albumin was approximately 0.21 ml/kg over 6 h). The animals in the comparator group received an equal volume of vehicle solution with 20% Albumin.

At the end of the protocol, the animals were euthanized with a lethal dose of sodium pentobarbitone (100 mg/kg, IV). The positions of the renal fiber-optic probes were confirmed at autopsy, and kidney biopsies were taken for histopathological assessment^23^.

### Randomization and blinding

Randomization was performed by the investigators using the online randomization module in Castor EDC at 24 h after sepsis induction. From this point forward, investigators conducting experiments and analyzing physiological data were aware of group allocation. Histopathological assessment was performed by a pathologist blinded to group allocation, and all physiological measurements were obtained through objective, automated recording systems.

### Data collection

A computer with a CED 1401 interface running a data acquisition system (Spike 2 Software, Cambridge Electronic Design, Cambridge, United Kingdom) continuously recorded analog signals (MAP, heart rate, CO, renal blood flow, RCP, RMP, PrcO_2,_ and RrmO_2_, temperature, and PuO_2_) at 100 Hz. Renal vascular conductance (RVC) was calculated as RBF/MAP. Stroke volume (SV) was calculated as CO/heart rate. We calculated the body surface area (BSA) as 0.09 × weight ^(0.67)^, and the cardiac index (CI) and stroke volume index (SVI) were calculated as the CO/BSA and SV/BSA, respectively. We recorded the hourly urine flow and collected 1-hourly urine samples at baseline and at 24, 26, 28, and 30 h after the induction of sepsis, corresponding to before the start of the intervention and 2, 4, and 6 h after the intervention, respectively. Urine samples were collected for measurement of creatinine and sodium concentrations and subsequent analysis of renal excretory function. Arterial and renal venous blood samples were collected at baseline, just prior to the infusion of *E. coli*, and subsequently at 24, 26, 28, and 30 h of sepsis for the measurement of blood oximetry (ABL System 625, Radiometer Medical, Copenhagen, Denmark), creatinine, and glucose. The occurrence of AKI was based on the “Kidney Disease: Improving Global Outcomes (KDIGO)” clinical criteria; stage 1 AKI is characterized by a > 1.5-fold increase in plasma creatinine or oliguria of 0.5 ml/kg/h for > 6 h.

### Statistical analysis

Data are reported as the mean ± SD, and between-group differences are reported as the difference with a 95% confidence interval (95% CI). All outcome measurements are reported as the absolute changes from the start of the intervention period^24^. The data were analyzed via repeated-measures analysis of variance (ANOVA) with the factors intervention (P_Intervention_: vehicle or GLP-1) and time (P_Time_). This approach accounts for any baseline differences between groups at randomization. The treatment effect was evaluated on the basis of the interaction term P_Intervention*Time_. Given its critical role in the development of AKI, we defined renal medullary tissue oxygenation as the primary outcome^19^. On the basis of previous studies, detecting a 50% improvement in medullary tissue oxygenation (mean pO_2_ = 10 ± 9) with 90% power and α = 0.05 required a sample size of eight sheep per group^19^. The histological assessment of kidney tissues was performed according to the semiquantitative histological scoring system^25^ by a pathologist blinded to group allocation and analyzed with Fisher’s exact test. Statistical analysis was performed via GraphPad PRISM 6.0 (GraphPad Software, La Jolla, CA). All variables were assessed for normality and log-transformed where appropriate. A two-sided *P* value less than or equal to 0.05 was considered statistically significant without correction for multiple comparisons.

## Results

During the 24-h period of sepsis induction, the sheep in both groups developed changes in hemodynamics and organ dysfunction consistent with sepsis, including hyperdynamic circulation with reductions in blood pressure, arterial oxygenation, and renal medullary oxygenation. Creatinine clearance and urine output decreased as plasma creatinine increased, which is consistent with the development of acute kidney injury (Table 1). Despite randomization, numerical differences in some renal parameters were present between groups at 24 h (Table 1), likely reflecting inter-individual variability in the renal response to sepsis. However, systemic markers of sepsis severity (hemodynamics, oxygenation, lactate, and AKI incidence) were comparable between groups at this timepoint. Supplementary Data S1 (Table and Figures) includes complete datasets showing the actual measured values at all time points. No animals died during the 30-h period after the infusion of *E. coli* commenced. No animals were excluded from the analysis.

**Table 1:** Changes in systemic hemodynamics, global and regional kidney perfusion, oxygenation, and renal function from baseline (premorbid) to 24 h of gram-negative sepsis in nonanesthetized sheep in both treatment groups.

Systemic and renal variables	GLP-1 group (*n* = 8)		Vehicle group (*n* = 8)	
	Before sepsis (0 h)	End sepsis (23.5 h)	Before sepsis (0 h)	End sepsis (23.5 h)
Body weight	38.9 ± 4.5		39.4 ± 3.5	
Mean arterial pressure (MAP, mmHg)	90 ± 14	75 ± 7.9*	88 ± 13	83 ± 9.9*
Heart rate (HR, bpm)	75 ± 5.8	106 ± 21*	77 ± 16	138 ± 28*
Cardiac output (CO, l/min)	4.0 ± 0.4	5.3 ± 0.8*	4.6 ± 0.7	5.8 ± 1.9*
Systemic vascular resistance (SVR, mmHg/ml/min)	22 ± 4.4	15 ± 2.9*	20 ± 2.8	17 ± 11*
Urine Output (UO, ml/kg/h)	1.5 ± 0.6	0.9 ± 0.9*	1.3 ± 0.6	0.9 ± 0.3*
Urinary Oxygenation (PuO_2_, mmHg)	37 ± 12	23 ± 14*	27 ± 11	16 ± 16*
AKI grade 1 (KDIGO criteria**)	0/8	7/8*	0/8	7/8*
Creatinine clearance (ml/min)	86 ± 31	52 ± 14*	85 ± 14	58 ± 26*
Plasma creatinine (µmol/l)	65.5 ± 11.3	109.3 ± 34.1*	63.6 ± 8.7	101.4 ± 16.4*
Plasma lactate (mmol/l)	0.6 ± 0.1	1.1 ± 0.5*	0.5 ± 0.1	1.4 ± 0.6*
Arterial oxygen tension (PaO_2,_ mmHg)	106 ± 18	95 ± 9.3*	95 ± 9.5	91 ± 9.3
Renal blood flow (RBF, ml/min)	278 ± 73	285 ± 116	335 ± 75	397 ± 64
Renal oxygen delivery (RDO_2_, ml O_2_/min)	37 ± 11	34 ± 15*	40 ± 9.4	52 ± 10*
Renal oxygen consumption (RVO_2_, ml O_2_/min)	5.0 ± 1.4	4.1 ± 1.6*	5.2 ± 1.9	4.6 ± 0.6*
Renal cortical tissue perfusion (RCP, BPU)	2067 ± 773	1367 ± 1231	1861 ± 902	2406 ± 1105
Renal medullary tissue perfusion (RMP, BPU)	1211 ± 858	512 ± 371*	817 ± 358	563 ± 279*
Renal cortical oxygen tension (PrcO_2_, mmHg)	24 ± 9.0	30 ± 19*	32 ± 11	45 ± 6.1*
Renal medullary oxygen tension (PrmO_2_, mmHg)	30 ± 12.7	26 ± 19	35 ± 15	26 ± 19

*P < 0.05 comparison of measurements before with end of sepsis. **KDIGO Stage 1 AKI: ≥1.5× creatinine increase OR urine output < 0.5 ml/kg/h for > 6 h.

### Systemic hemodynamic function

**Fig. 2:**
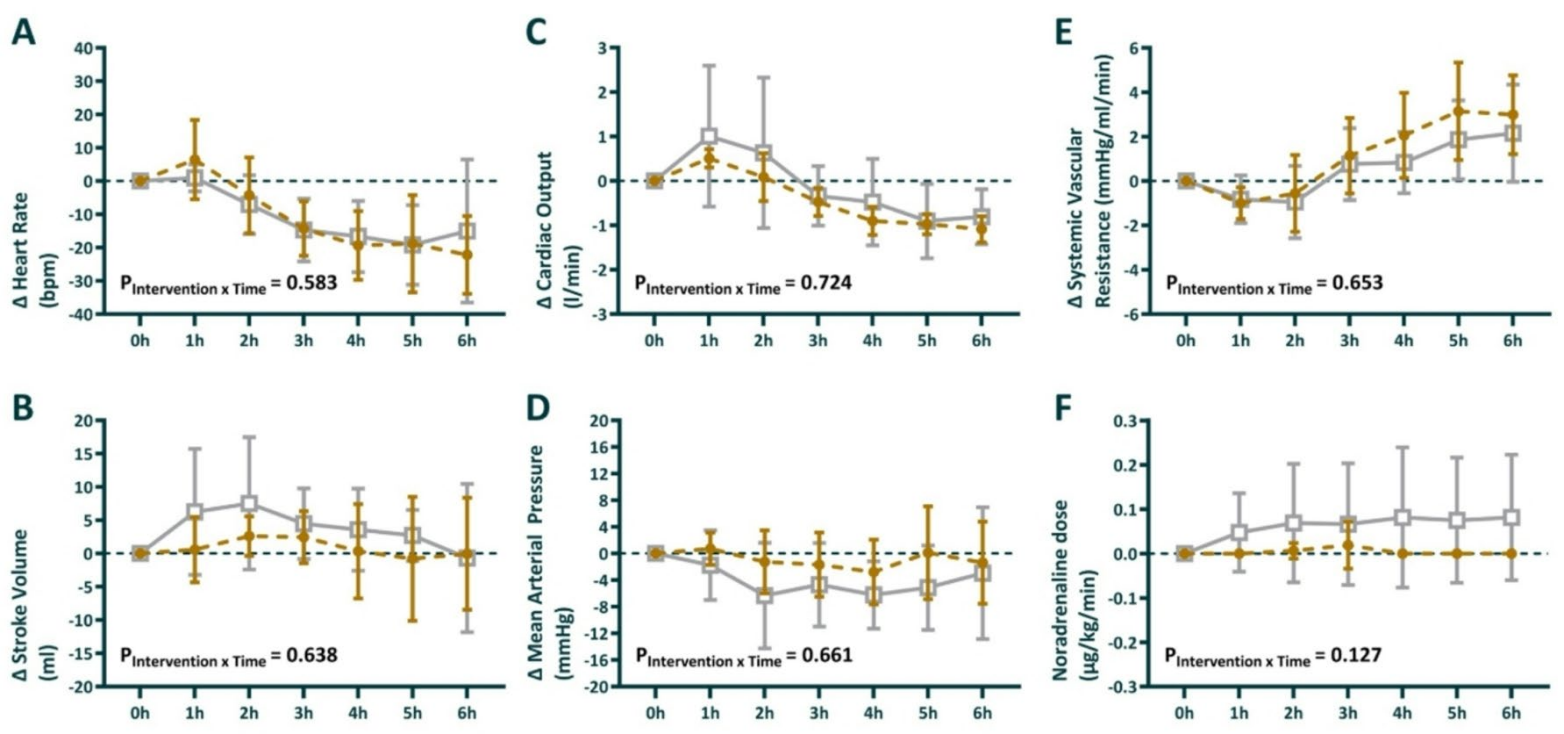
Absolute change in heart rate (**A**), stroke volume (**B**), cardiac output (**C**), mean arterial pressure (**D**), systemic vascular resistance (**E**) and dose of noradrenaline required (**F**) during the intervention period. 0 h is the start of the intervention period, corresponding to 24 h of sepsis. Sheep randomized to intravenous infusion of 3.6 pmol/kg/min GLP-1 (*n* = 8) are presented as closed gold circles and those to vehicle solution (*n* = 8) as open gray squares. Values are mean ± sd changes from the end of the intervention. The P-values are the outcome of the treatment × time interaction term from a two-way repeated-measures ANOVA, comparing the pre-treatment value with the 6 time points in the intervention period.

After 24 h of *E. coli* infusion, the sheep developed a hypotensive hyperdynamic circulatory state, with an increased heart rate and a reduced stroke volume and systemic vascular resistance (Table 1). During sepsis induction, arterial lactate increased from 0.48 ± 0.10 to 1.35 ± 0.63 mmol/L. Following fluid resuscitation, the intervention period did not result in significant between-group differences in systemic hemodynamics, and the interaction effect P_intervention*time_ did not reach statistical significance for any of these systemic hemodynamic parameters when GLP-1 infusion was compared with vehicle. (Fig. 2A–F).

### Kidney function

**Fig. 3:**
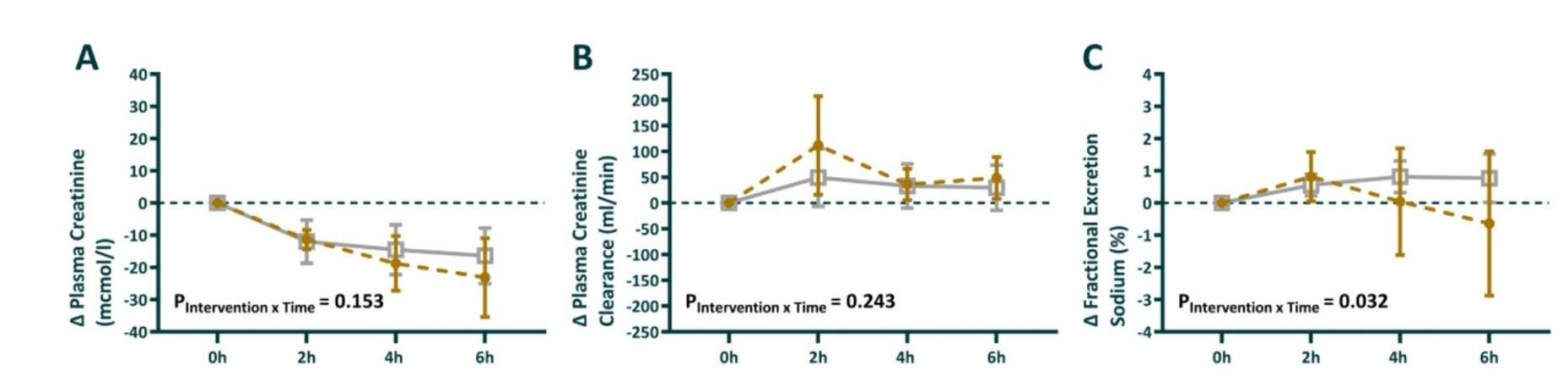
Absolute change in Plasma creatinine (**A**), creatinine clearance (**B**), and fractional excretion of sodium (**C**) during the intervention period. 0 h is the start of the intervention period, corresponding to 24 h of sepsis. Sheep randomized to intravenous infusion of 3.6 pmol/kg/min GLP-1 (*n* = 8) are presented as closed gold circles and those to vehicle solution (*n* = 8) as open gray squares. Values are mean ± sd changes from the end of the intervention. The P-values are the outcome of the treatment × time interaction term from a two-way repeated-measures ANOVA, comparing the pre-treatment value with the 3 time points in the 6 h intervention period.

At 24 h after the commencement of *E. coli* infusion, 7 out of 8 sheep in each group had developed stage 1 AKI. During the induction of sepsis, plasma creatinine increased, and creatinine clearance decreased (Table 1). During the intervention period, plasma creatinine decreased, and creatinine clearance increased without any differences between the groups (Fig. 3A + B). While the fractional excretion of sodium was stable in the vehicle group, it decreased during the intervention period in the GLP-1-treated group, with P_intervention*time_ = 0.032 (Fig. 3C).

### Glycemia

Blood glucose levels remained stable and within normal ranges during the study period (0 h = 3.0 ± 0.45, 24 h = 2.9 ± 1.5, 30 h = 3.3 ± 1.6 mmol/l). Normal ovine glycemia ranges from 1.4 to 3.6 mmol/l^26,27^. Glucose measurements are summarized per group in Supplementary Data S1 (Table and Figures), without any between-group differences.

### Global kidney perfusion and oxygen handling

**Fig. 4:**
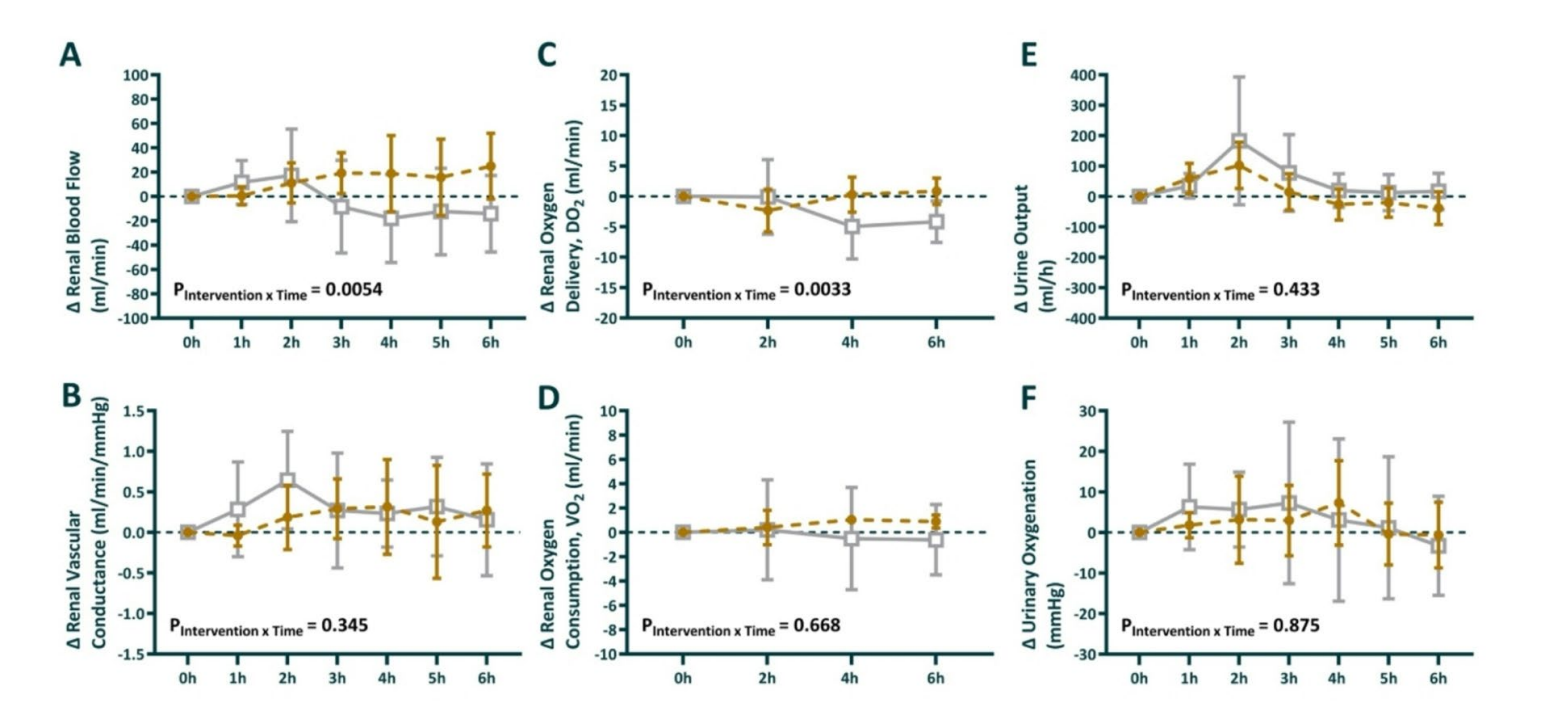
Absolute change in renal blood flow (**A**), renal vascular conductance (**B**), renal oxygen delivery (**C**), renal oxygen consumption (**E**), urine output (**E**), and, urine oxygenation (**F**) during the intervention period. 0 h is the start of the intervention period, corresponding to 24 h of sepsis. Sheep randomized to intravenous infusion of 3.6 pmol/kg/min GLP-1 (*n* = 8) are presented as closed gold circles and those to vehicle solution (*n* = 8) as open gray squares. Values are mean ± sd changes from the end of the intervention. The P-values are the outcome of the treatment × time interaction term from a two-way repeated-measures ANOVA, comparing the pre-treatment value with the 6 time points in the intervention period.

During the induction of sepsis, renal blood flow increased by 62 ± 30 ml/min in the GLP-1 group vs. 7 ± 46 ml/min in the vehicle group (Table 1). During the intervention (24–30 h), mean change in renal blood flow was + 13 in the GLP-1 vs. -3.4 in the vehicle group, difference (95% CI) = 16 (− 7.4–40) *P* = 0.16. Although P_intervention*time_ = 0.0054 (Fig. 4A). Renal vascular conductance increased during sepsis (0.64 ± 0.59 ml/min/mmHg in GLP-1 group vs. 1.2 ± 0.17 ml/min/mmHg in vehicle group) during the intervention period, this remained relatively stable in both groups mean change 0.16 vs. 0.27 difference (95% CI) = − 0.1 (− 0.57–0.35) *P* = 0.61. Renal oxygen delivery decreased during the sepsis period in the GLP-1 group (− 2.3 ± 1.4 ml/min) while it increased in the vehicle group (+ 12 ± 3.6). From the start of intervention, Renal oxygen delivery remained stable in the GLP-1 group compared to a decrease in the vehicle group (− 0.3 vs. − 2.3 ml/min), difference (95% CI) = 2.0 (− 1.3–5.4), *P* = 0.21 (Fig. 4C). However, the interaction term for P_intervention*time_ = 0.033. In contrast, renal oxygen consumption decreased during the sepsis period, but remained unaffected by the intervention, without between-group differences (Fig. 4D). Urine output (Fig. 4E) decreased between baseline and the end of sepsis induction (Table 1). During the intervention period, urine output remained relatively stable, without significant differences between the groups. Similarly, urinary oxygenation did not differ significantly between the groups (Fig. 4F).

### Intrarenal perfusion and oxygenation

**Fig. 5:**
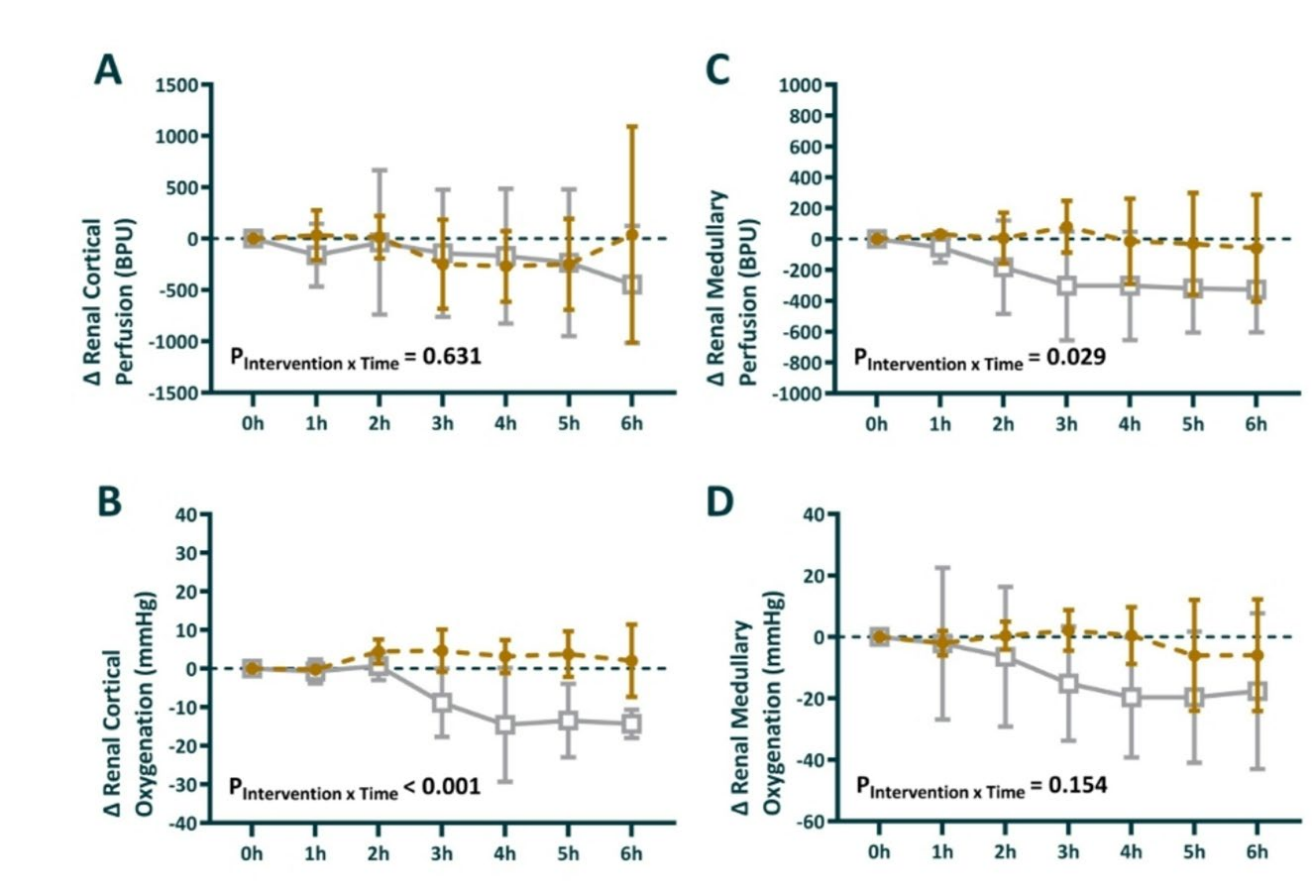
Absolute change in renal cortical perfusion (**A**) and oxygenation (**B**), and renal medullary perfusion (**C**) and oxygenation (**D**) during the intervention period. 0 h is the start of the intervention period, corresponding to 24 h of sepsis. Sheep randomized to intravenous infusion of 3.6 pmol/kg/min GLP-1 (*n* = 8) are presented as closed gold circles and those to vehicle solution (*n* = 8) as open gray squares. Values are mean ± sd changes from the end of the intervention. The P-values are the outcome of the treatment × time interaction term from a two-way repeated-measures ANOVA, comparing the pre-treatment value with the 6 time points in the intervention period.

During sepsis, renal cortical perfusion decreased by -737 ± 479 BPU in the GLP-1 group vs. an increase of 730 ± 580 BPU in the vehicle group (Table 1). During the intervention (24–30 h), mean change in renal cortical perfusion was − 97 BPU in the GLP-1 vs. − 169 BPU in the vehicle group, difference (95% CI) = 72 (− 299–444) *P* = 0.67 (Fig. 5A).

Renal cortical oxygenation increased during sepsis by 5.0 ± 5.6 mmHg in the GLP-1 group vs. 13.3 ± 3.4 mmHg in the vehicle group (Table 1). During the intervention (24–30 h), mean change in renal cortical oxygenation was + 2.5 mmHg in the GLP-1 group vs. − 7.2 mmHg in the vehicle group, difference (95% CI) = 9.7 (5.2–14.4) *P* = 0.0008 (Fig. 5B).

During sepsis, renal medullary perfusion decreased by − 700 ± 266 BPU in the GLP-1 group vs. − 263 ± 113 BPU in the vehicle group (Table 1). The GLP-1 group maintained renal medullary perfusion during the intervention period, whereas this decreased in the placebo group + 1.7 BPU vs. − 213 BPU, difference (95% CI) = 214 (− 21–450) *P* = 0.07, though the interaction term P_Intervention*Time_ = 0.029 (Fig. 5C).

We observed a similar profile in renal medullary oxygenation, although the between-group difference was not statistically significant (P_Intervention*Time_ = 0.154). Following an initial decrease in renal medullary oxygenation, − 5.3 ± 4.4 mmHg in the GLP-1 group vs. − 6.1 ± 9.3 mmHg in the vehicle group (Table 1). Renal medullary remained stable during the intervention period in the GLP-1 group and decreased further in the placebo group − 1.6 mmHg vs. − 11.5 mmHg, difference (95% CI) = 9.9 (− 6.8–26.7) *P* = 0.21 (Fig. 5D).

#### Renal histopathology

Histopathological examination of renal biopsies revealed diffuse tubular injury in three sheep in the GLP-1 group and one in the vehicle group (Table 2). In addition, focal tubular injury was present in one sheep in each group. The incidence of tubular injury was not significantly different between the groups (4/8 vs. 2/8, respectively, *P* = 0.6). Inflammatory changes were present in three of the sheep in each treatment group. Tubular casts were observed in 4/8 of the GLP-1-treated group and in 5/8 of the vehicle-treated group (*P* = 0.32). Between-group comparisons of inflammatory changes and the presence of tubular casts revealed no significant differences between the GLP-1 and vehicle groups (Supplementary Data S2, Table).

**Table 2:** Renal pathological changes in GLP-1 and vehicle-treated groups.

	GLP-1 (*n* = 8)								Vehicle (*n* = 8)							
	G1	G2	G3	G4	G5	G6	G7	G8	V1	V2	V3	V4	V5	V6	V7	V8
Tubular injury	+	0	++	++	++	0	0	0	0	0	+	0	0	0	++	0
Interstitial inflammation	0	0	++	++	++	0	0	0	++	0	+	0	0	0	++	0
Tubular casts	0	0	++	++	++	0	+	0	0	0	+	++	0	++	+	+

Zero (0), no histological renal tubular injury, inflammation, or tubular casts; (+) = mild or focal histological renal tubular injury, inflammation, or tubular casts; (++) = significant histological renal tubular injury, inflammation, or tubular casts.

## Discussion

In an ovine model of gram-negative SA-AKI, we studied the acute effects of GLP-1 on systemic hemodynamics, renal function, and intrarenal perfusion and oxygenation. GLP-1 infusion did not result in a significant difference between groups in the primary endpoint of renal medullary oxygenation or kidney function. However, during the intervention period, GLP-1-treated sheep showed divergent trajectories from vehicle-treated sheep for several secondary endpoints. Specifically, renal blood flow continued to increase in the GLP-1 group, whereas it declined in the vehicle group. Renal cortical oxygenation and renal medullary perfusion increased in the GLP-1 group while decreasing in the vehicle group.

GLP-1 treatment improved renal medullary perfusion but did not achieve statistical significance for medullary oxygenation (*p* = 0.115), despite similar directional profiles for both parameters. This discrepancy may reflect the study being underpowered for the modest effect size observed, greater measurement variability in oxygenation compared with perfusion, or the possibility that improved perfusion was accompanied by increased local oxygen consumption. The clinical relevance of improved medullary perfusion, even without statistically significant improvement in oxygenation, remains uncertain and warrants further investigation in larger studies.

### Relationship to previous studies

Clinical research on GLP-1 in critically ill patients has focused mainly on its effects on glycemia, glucose-regulating hormones, and gastric emptying^10^. These studies revealed that GLP-1 significantly attenuated hyperglycemia in the ICU but also delayed gastric emptying^10^. Currently, the literature concerning GLP-1 treatment is conflicting regarding the associated risk of AKI. A meta-analysis including 11 large (> 500 participants) randomized trials studying the effects of GLP-1 receptor agonists on kidney outcomes reported 16% lower rates of kidney failure (HR = 0.84, 95% CI = 0.72–0.99)^8^. In addition, real-world data analysis of the outcomes of GLP-1 RA users revealed significantly lower incidences of major adverse kidney events (HR = 0.66, 95% CI = 0.60–0.73)^28^. In contrast, concerns have been raised that GLP-1 RAs could be associated with kidney injury on the basis of case reports of acute interstitial nephritis following GLP-1 RA treatment, likely in the context of reduced intake during illness^29^. While our study revealed improvements in renal blood flow, cortical oxygenation, and medullary perfusion, histopathology revealed tubular injury in four of the eight sheep in the GLP-1 group compared with two in the vehicle group. Therefore, GLP-1 treatment may have both beneficial and harmful effects on kidney health. How to interpret and balance these conflicting risks requires further research, which could also inform more individualized management on the basis of the context of patient characteristics or (intercurrent) comorbidities.

Another important difference between our study and the data from real-world databases or long-term follow-up trials is the moment of treatment initiation^8,28^. Chronic GLP-1 RA use results in effective plasma concentrations before an index event, potentially leading to AKI (such as critical illness or surgery). In this study, GLP-1 was administered after 24 h of established sepsis when 14/16 sheep had already met the KDIGO criteria for Stage 1 AKI. While in line with the clinical practice of the ICU, this illustrates a critical difference between preventive effects from chronic treatment and the attempt to treat after the moment of recognition of AKI.

The effects of GLP-1 treatment on renal oxygenation and perfusion have been studied previously in animals and healthy human volunteers^9^. Studies in rodents demonstrated the presence of GLP-1 receptors in the renal vasculature, which were found to induce preglomerular vasodilation, reduce renal vascular resistance, and increase renal blood flow^30,31^. In our study, we observed increased renal blood flow with GLP-1 treatment. Renal vascular conductance showed a parallel pattern, consistent with the mathematical relationship (RVC = RBF/MAP) in the context of stable blood pressure, though with greater variability. In humans, magnetic resonance imaging demonstrated that GLP-1 infusion improved renal cortical and medullary perfusion, although renal blood flow was unaffected^9^. This study also demonstrated that GLP-1 infusion attenuated the decline in cortical oxygenation observed in vehicle-treated sheep, although not of the medulla^9^. Notably, our study included an ovine model and the context of sepsis induction, resulting in increased renal blood flow with reduced renal medullary perfusion and oxygenation. In our model, GLP-1 increased renal blood flow, renal cortical oxygenation, and medullary perfusion. While oxygenation of the renal medulla did not improve, augmenting renal medullary perfusion in a state of compromised perfusion could have contributed to kidney protection^32^. Regardless, in our study, the difference in medullary perfusion did not translate into improved oxygenation or renal function.

A modest increase in the resting heart rate during GLP-1 treatment has been extensively described in animal and human studies^33,34^. This seems to be mediated through direct action on the sinus node^34^. However, these effects were demonstrated during periods of dominant parasympathetic activity^33^. The lack of increase in heart rate following GLP-1 treatment in our study might be explained by the context of sepsis-induced sympathetic activation, which elevated heart rate and cardiac output.

### Strengths and limitations

We studied the renal effects of GLP-1 in an established, reproducible, clinically relevant large animal model of sepsis-associated AKI^12,15,18,20,21^. The model closely resembles cardiovascular and renal physiology during the early stage of sepsis^12,15,18,20,21^. The study methodology was designed with clinical practice in mind. Sepsis was induced over 24 h without other interventions; then, at a time analogous to hospital presentation, treatment was initiated, following per-protocol fluid resuscitation. Group allocation was based on randomization, and histopathological analyses were performed by an experienced pathologist blinded to treatment group allocation. However, our treatment period was limited to 6 h (from 24 to 30 h of sepsis). Therefore, we cannot comment on the effects and outcomes of longer continuous treatment. Assessing the state and severity of sepsis in an animal model can be difficult, especially compared with clinical experience. However, the degree of organ dysfunction reflected by increases in cardiac output and lactate levels or decreases in arterial oxygenation and urine output is comparable to that reported in previous studies and reflects a state of early sepsis and the development of organ dysfunction. However, despite randomization, differences in some renal hemodynamic parameters were observed between groups at 24 h, with less pronounced changes in some parameters during sepsis induction compared to previous studies from our laboratory. This biological heterogeneity underscores the importance of cautious interpretation in relation to clinical practice. While systemic sepsis severity remained comparable between groups, this variability is an inherent limitation of large animal research. Our statistical approach of analyzing changes from the start of the intervention (24 h) was designed to assess treatment effects on the established disease state, which is the clinically relevant question. Nevertheless, we acknowledge that baseline differences at randomization may influence treatment responses and warrant cautious interpretation of our findings.

Comparison to clinical practice is limited by, the experimental induction of sepsis through the intravenous administration of *E. coli*, in contrast to the primary organ focus of sepsis. Unlike in clinical practice, we studied young female sheep without known comorbidities. Our sample size calculation assumed a 50% reduction in medullary oxygenation during sepsis based on previous studies, but the current cohort showed a more modest reduction (~ 20%). While adequately powered to detect large treatment effects from severely compromised baselines, the study may have been underpowered to detect modest improvements from less severely compromised baselines.

## Conclusion

In a large animal model of live gram-negative sepsis-associated AKI, we found no significant improvement in kidney function or renal medullary oxygenation following GLP-1 infusion. However, GLP-1 treatment was associated with divergent renal hemodynamic trajectories during the intervention period, with GLP-1-treated sheep showing continued increases in renal blood flow, oxygen delivery, cortical oxygenation, and medullary perfusion, whereas these parameters declined in vehicle-treated sheep.

## Supplementary Information

Below is the link to the electronic supplementary material.

Supplementary Material 1

Supplementary Material 2

## Data Availability

Data are available upon request to the corresponding author.
