# Supplementary material for: Effects of glucagon-like peptide-1 on systemic hemodynamics, kidney function, and intrarenal oxygenation in sheep with sepsis-associated acute kidney injury
Source: Sci Rep. 2025 Dec 24;16:3250. doi: 10.1038/s41598-025-33109-0 (PMC12830701; doi:10.1038/s41598-025-33109-0)

**Sheet**	Heart Rate
**Graph**	GLP-1 vs Vehicle
**Title**	Heart Rate - GLP-1 vs Vehicle
**Legend**	HR (bpm)

	GLP-1												Vehicle											
**Time (h)**	**Gamgee**	**Took**	**Simba**	**Wiggins**	**Ratcliffe**	**Bashful**	**Sneezy**	**Shorty**	**MEAN**	**SD**	**N**	**SEM**	**Timon**	**Kocoum**	**Percy**	**Ben 2**	**Zazu**	**CPB101**	**Dumbo**	**Doc**	**MEAN**	**SD**	**N**	**SEM**
**Baseline**	73.44	75.41	83.38	72.91	68.35	66.45	81.34	75.39	74.58	5.77	8.00	2.04	61.98	67.89	73.93	79.76	63.81	70.08	90.49	110.49	77.30	16.26	8.00	5.75
**End of Sepsis**	121.58	94.75	98.19	110.01	69.18	93.07	118.43	139.66	105.61	21.57	8.00	7.62	127.18	158.39	117.91	101.21	111.33	183.53	150.90	155.70	138.27	28.17	8.00	9.96
**25**	111.91	108.56	117.65	114.24	126.50	96.59	128.35	132.89	117.09	11.91	8.00	4.21	132.70	164.72	127.09	108.20	101.53	182.21	161.88	169.48	143.48	30.13	8.00	10.65
**26**	94.97	92.26	128.34	100.19	80.68	91.83	118.42	143.99	106.34	21.65	8.00	7.66	129.87	158.58	107.64	110.35	98.64	182.06	153.31	142.94	135.42	28.96	8.00	10.24
**27**	90.94	96.92	105.05	103.95	68.59	82.77	102.54	120.01	96.35	15.66	8.00	5.54	124.87	151.37	101.53	90.90	100.61	180.85	133.85	138.13	127.76	30.03	8.00	10.62
**28**	85.80	83.41	103.52	95.49	74.95	88.49	94.64	103.93	91.28	10.04	8.00	3.55	119.91	143.36	122.37	84.04	92.46	183.69	122.33	138.51	125.83	31.02	8.00	10.97
**29**	82.40	84.17	95.84	92.28	73.17	104.89	102.25	99.19	91.77	10.99	8.00	3.88	124.86	150.67	108.48	75.47	89.61	182.57	122.53	132.12	123.29	33.78	8.00	11.94
**30**	80.87	80.60	90.50	101.44	77.86	81.00	100.70	94.54	88.44	9.61	8.00	3.40	122.58	153.54	114.34	130.87	84.72	178.29	109.56	125.92	127.48	28.34	8.00	10.02

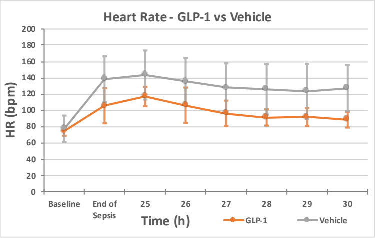

**Sheet**	Stroke Volume
**Graph**	GLP-1 vs Vehicle
**Title**	Stroke Volume - GLP-1 vs Vehicle
**Legend**	SV (ml)

	GLP-1												Vehicle											
**Time (h)**	**Gamgee**	**Took**	**Simba**	**Wiggins**	**Ratcliffe**	**Bashful**	**Sneezy**	**Shorty**	**MEAN**	**SD**	**N**	**SEM**	**Timon**	**Kocoum**	**Percy**	**Ben 2**	**Zazu**	**CPB101**	**Dumbo**	**Doc**	**MEAN**	**SD**	**N**	**SEM**
**Baseline**	59.18	58.17	56.47	48.12	54.69	59.33	48.65	47.80	54.05	5.08	8.00	1.80		78.79	67.37	58.32	57.95		43.18	47.67	58.88	12.98	6.00	5.30
**End of Sepsis**	48.71	58.74	54.47	53.03	51.44	55.60	51.67	34.02	50.96	7.48	8.00	2.64		15.05	58.76	69.51	52.02		34.45	48.04	46.31	19.22	6.00	7.85
**25**	60.22	68.14	59.21	56.43	41.30	60.53	52.82	40.99	54.95	9.55	8.00	3.38		62.54	61.24	75.96	56.81		39.65	50.66	57.81	12.21	6.00	4.98
**26**	63.07	67.21	57.55	58.42	50.25	60.50	54.34	41.59	56.62	7.97	8.00	2.82		63.36	68.52	76.24	53.20		40.15	53.06	59.09	12.89	6.00	5.26
**27**	65.34	64.55	57.37	55.80	51.42	58.33	53.63	42.63	56.13	7.30	8.00	2.58		45.55	67.87	74.12	50.18		43.33	55.26	56.05	12.44	6.00	5.08
**28**	65.89	64.17	57.41	52.83	43.10	51.09	54.53	44.88	54.24	8.18	8.00	2.89		50.64	58.28	71.41	51.95		43.56	55.07	55.15	9.37	6.00	3.83
**29**	66.91	65.21	55.96	54.34	43.02	41.99	56.62	42.91	53.37	9.91	8.00	3.50		40.34	60.31	71.89	53.08		45.39	54.72	54.29	11.15	6.00	4.55
**30**	69.57	65.49	53.79	48.82	39.90	57.67	54.83	42.50	54.07	10.33	8.00	3.65		40.87	62.31	50.90	52.83		45.53	52.83	50.88	7.31	6.00	2.98

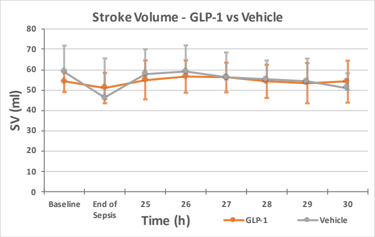

**Sheet**	Cardiac Output
**Graph**	GLP-1 vs Vehicle
**Title**	Cardiac Output - GLP-1 vs Vehicle
**Legend**	CO (l/min)

	GLP-1												Vehicle											
**Time (h)**	**Gamgee**	**Took**	**Simba**	**Wiggins**	**Ratcliffe**	**Bashful**	**Sneezy**	**Shorty**	**MEAN**	**SD**	**N**	**SEM**	**Timon**	**Kocoum**	**Percy**	**Ben 2**	**Zazu**	**CPB101**	**Dumbo**	**Doc**	**MEAN**	**SD**	**N**	**SEM**
**Baseline**	4.35	4.39	4.71	3.51	3.74	3.94	3.96	3.60	4.02	0.42	8.00	0.15		5.35	4.98	4.65	3.70		3.91	5.27	4.64	0.70	6.00	0.29
**End of Sepsis**	5.91	5.57	5.35	5.83	3.51	5.17	6.12	4.73	5.28	0.84	8.00	0.30		2.38	6.90	7.04	5.77		5.20	7.48	5.79	1.88	6.00	0.77
**25**	6.74	7.40	6.97	6.45	5.22	5.85	6.78	5.45	6.36	0.77	8.00	0.27		10.30	7.78	8.22	5.77		6.42	8.59	7.85	1.62	6.00	0.66
**26**	5.99	6.20	7.39	5.85	4.05	5.56	6.44	5.99	5.93	0.94	8.00	0.33		10.05	7.38	8.41	5.25		6.16	7.59	7.47	1.69	6.00	0.69
**27**	5.94	6.26	6.03	5.80	3.53	4.83	5.50	5.12	5.37	0.89	8.00	0.31		6.89	6.89	6.74	5.05		5.80	7.63	6.50	0.92	6.00	0.38
**28**	5.65	5.35	5.94	5.04	3.23	4.52	5.16	4.66	4.95	0.84	8.00	0.30		7.26	7.13	6.00	4.80		5.33	7.63	6.36	1.15	6.00	0.47
**29**	5.51	5.49	5.36	5.01	3.15	4.40	5.79	4.26	4.87	0.88	8.00	0.31		6.08	6.54	5.43	4.76		5.56	7.23	5.93	0.88	6.00	0.36
**30**	5.63	5.28	4.87	4.95	3.11	4.67	5.52	4.02	4.76	0.84	8.00	0.30		6.27	7.12	6.66	4.48		4.99	6.65	6.03	1.05	6.00	0.43

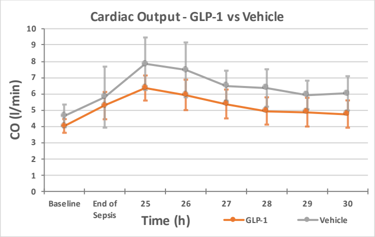

**Sheet**	Mean Arterial Pressure
**Graph**	GLP-1 vs Vehicle
**Title**	Mean Arterial Pressure - GLP-1 vs Vehicle
**Legend**	MAP (mmHg)

	GLP-1												Vehicle											
**Time (h)**	**Gamgee**	**Took**	**Simba**	**Wiggins**	**Ratcliffe**	**Bashful**	**Sneezy**	**Shorty**	**MEAN**	**SD**	**N**	**SEM**	**Timon**	**Kocoum**	**Percy**	**Ben 2**	**Zazu**	**CPB101**	**Dumbo**	**Doc**	**MEAN**	**SD**	**N**	**SEM**
**Baseline**	88.76	89.95	101.52	85.35	118.82	93.52	79.98	85.18	92.88	12.28	8.00	4.34	80.34	85.27	83.42	95.41	81.88	80.05	82.99	117.24	88.32	12.66	8.00	4.48
**End of Sepsis**	67.71	72.99	93.20	75.17	69.45	75.96	70.46	74.58	74.94	7.94	8.00	2.81	70.09	82.08	86.82	90.86	78.61	82.61	73.77	91.71	82.07	7.72	8.00	2.73
**25**	65.48	73.80	89.16	74.21	79.12	79.99	75.92	76.52	76.77	6.69	8.00	2.36	70.14	78.82	79.66	101.69	79.13	86.05	64.00	67.02	78.32	12.00	8.00	4.24
**26**	71.21	73.19	84.81	74.25	72.82	72.35	77.00	72.96	74.82	4.38	8.00	1.55	65.23	75.67	84.00	98.84	73.72	80.82	52.46	59.29	73.75	14.75	8.00	5.22
**27**	71.34	70.26	84.60	74.02	75.04	81.74	66.37	71.82	74.40	6.05	8.00	2.14	66.69	69.50	89.88	94.80	73.18	79.03	62.42	67.54	75.38	11.62	8.00	4.11
**28**	69.48	68.55	83.17	71.67	72.44	82.27	66.25	72.47	73.29	6.20	8.00	2.19	65.34	75.92	78.49	83.15	72.42	81.50	66.22	67.72	73.85	6.98	8.00	2.47
**29**	75.19	69.50	83.69	77.88	74.06	98.37	70.22	70.71	77.45	9.67	8.00	3.42	69.06	67.77	81.37	89.97	73.93	78.98	72.21	66.24	74.94	8.05	8.00	2.85
**30**	74.68	73.93	82.59	69.67	73.58	83.22	67.87	72.04	74.70	5.55	8.00	1.96	70.06	71.01	85.87	78.49	80.45	81.18	79.31	70.67	77.13	5.85	8.00	2.07

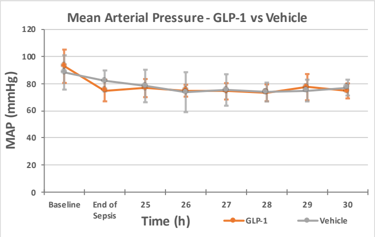

**Sheet**	Systemic Vascular Resistance
**Graph**	GLP-1 vs Vehicle
**Title**	Systemic Vascular Resistance - GLP-1 vs Vehicle
**Legend**	SVR (mmHg/ml/min)

	GLP-1												Vehicle											
**Time (h)**	**Gamgee**	**Took**	**Simba**	**Wiggins**	**Ratcliffe**	**Bashful**	**Sneezy**	**Shorty**	**MEAN**	**SD**	**N**	**SEM**	**Timon**	**Kocoum**	**Percy**	**Ben 2**	**Zazu**	**CPB101**	**Dumbo**	**Doc**	**MEAN**	**SD**	**N**	**SEM**
**Baseline**	20.42	17.03	21.56	24.33	31.78	21.15	20.21	23.63	22.52	4.36	8.00	1.54		15.94	16.75	20.51	22.14		21.24	22.26	19.81	2.77	6.00	1.13
**End of Sepsis**	11.46	13.12	17.45	12.90	19.78	14.68	11.52	15.76	14.58	2.95	8.00	1.04		39.43	12.61	13.00	13.11		14.20	12.26	17.44	10.80	6.00	4.41
**25**	9.72	9.98	12.80	11.51	15.14	13.68	11.20	14.05	12.26	1.97	8.00	0.70		7.65	10.23	12.37	13.72		9.97	7.81	10.29	2.42	6.00	0.99
**26**	11.89	11.80	11.48	12.69	17.96	13.02	11.97	12.18	12.87	2.11	8.00	0.75		7.53	11.39	11.75	14.05		8.52	7.82	10.18	2.62	6.00	1.07
**27**	12.01	11.23	14.04	12.76	21.28	16.93	12.07	14.04	14.29	3.33	8.00	1.18		10.08	13.04	14.07	14.50		10.76	8.85	11.88	2.31	6.00	0.94
**28**	12.29	12.81	13.99	14.21	22.43	18.20	12.84	15.54	15.29	3.45	8.00	1.22		10.46	11.01	13.86	15.08		12.43	8.88	11.95	2.29	6.00	0.93
**29**	13.64	12.66	15.60	15.53	23.53	22.34	12.13	16.62	16.50	4.26	8.00	1.51		11.15	12.44	16.58	15.54		12.98	9.16	12.98	2.75	6.00	1.12
**30**	13.27	14.01	16.97	14.07	23.68	17.81	12.29	17.93	16.25	3.69	8.00	1.30		11.32	12.05	11.78	17.98		15.90	10.62	13.27	2.95	6.00	1.21

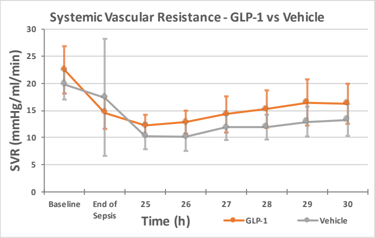

Supplementary Data S1 (Table + Figures). Data of absolute values for all sheep per group and graphical representation of trends over time. Starting at baseline, 24 hours of sepsis (start intervention) and 6h intervention period. Heart rate, stroke volume, cardiac output, mean arterial pressure, systemic vascular resistance, dose of noradrenaline required, renal blood flow, renal vascular conductance, urine output, and, urine oxygenation, renal cortical perfusion and oxygenation, renal medullary perfusion and oxygenation, plasma creatinine, creatinine clearance, fractional excretion of sodium, renal oxygen delivery, renal oxygen consumption, and glucose.

**Sheet**	Noradraneline dose
**Graph**	GLP-1 vs Vehicle
**Title**	Noradraneline dose - GLP-1 vs Vehicle
**Legend**	Noradraneline dose (mcg/l

	GLP-1												Vehicle											
**Time (h)**	**Gamgee**	**Took**	**Simba**	**Wiggins**	**Ratcliffe**	**Bashful**	**Sneezy**	**Shorty**	**MEAN**	**SD**	**N**	**SEM**	**Timon**	**Kocoum**	**Percy**	**Ben 2**	**Zazu**	**CPB101**	**Dumbo**	**Doc**	**MEAN**	**SD**	**N**	**SEM**
**Baseline**	0.00	0.00	0.00	0.00	0.00	0.00	0.00	0.00	0.00	0.00	8.00	0.00	0.00	0.00	0.00	0.00	0.00	0.00	0.00	0.00	0.00	0.00	8.00	0.00
**End of Sepsis**	0.00	0.00	0.00	0.00	0.00	0.00	0.00	0.00	0.00	0.00	8.00	0.00	0.00	0.00	0.00	0.00	0.00	0.00	0.05	0.05	0.01	0.02	8.00	0.01
**25**	0.00	0.00	0.00	0.00	0.00	0.00	0.00	0.00	0.00	0.00	8.00	0.00	0.00	0.00	0.00	0.00	0.00	0.00	0.20	0.18	0.05	0.09	8.00	0.03
**26**	0.00	0.00	0.00	0.00	0.00	0.00	0.05	0.00	0.01	0.02	8.00	0.01	0.00	0.00	0.00	0.00	0.00	0.00	0.35	0.20	0.07	0.13	8.00	0.05
**27**	0.00	0.00	0.00	0.00	0.00	0.00	0.15	0.00	0.02	0.05	8.00	0.02	0.00	0.00	0.00	0.00	0.00	0.00	0.38	0.15	0.07	0.14	8.00	0.05
**28**	0.00	0.00	0.00	0.00	0.00	0.00	0.00	0.00	0.00	0.00	8.00	0.00	0.00	0.05	0.00	0.00	0.00	0.00	0.45	0.15	0.08	0.16	8.00	0.06
**29**	0.00	0.00	0.00	0.00	0.00	0.00	0.00	0.00	0.00	0.00	8.00	0.00	0.00	0.05	0.00	0.00	0.00	0.00	0.40	0.15	0.08	0.14	8.00	0.05
**30**	0.00	0.00	0.00	0.00	0.00	0.00	0.08	0.00	0.01	0.03	8.00	0.01	0.00	0.10	0.00	0.00	0.00	0.00	0.40	0.15	0.08	0.14	8.00	0.05

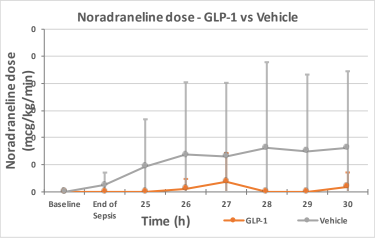

**Sheet**	Renal Blood Flow
**Graph**	GLP-1 vs Vehicle
**Title**	Renal Blood Flow - GLP-1 vs Vehicle
**Legend**	RBF (ml/min)

	GLP-1												Vehicle											
**Time (h)**	**Gamgee**	**Took**	**Simba**	**Wiggins**	**Ratcliffe**	**Bashful**	**Sneezy**	**Shorty**	**MEAN**	**SD**	**N**	**SEM**	**Timon**	**Kocoum**	**Percy**	**Ben 2**	**Zazu**	**CPB101**	**Dumbo**	**Doc**	**MEAN**	**SD**	**N**	**SEM**
**Baseline**	254.67	162.28	288.45	361.56		342.73	212.44	324.99	278.16	72.71	7.00	27.48	317.27	260.02	273.82	361.96	277.17	387.66		467.62	335.07	75.40	7.00	28.50
**End of Sepsis**	294.33	300.40	101.50			446.42	223.31	348.62	285.76	116.54	6.00	47.58	397.49	346.97	383.94	466.52	329.04	500.02		353.08	396.72	64.12	7.00	24.23
**25**	302.45	271.57	114.31			452.21	210.32	355.46	284.38	116.72	6.00	47.65	420.66	398.93	370.89	443.04	327.46	568.86		573.93	443.40	94.84	7.00	35.85
**26**	286.78	323.88	110.18			452.75	228.29	367.46	294.89	118.02	6.00	48.18	415.04	396.36	387.59	477.83	331.35	624.23		511.73	449.16	97.47	7.00	36.84
**27**	318.33	329.16	128.73			457.30	210.05	374.54	303.02	117.35	6.00	47.91	418.19	413.52	363.37	377.07	312.44	560.03		519.18	423.40	87.59	7.00	33.11
**28**	313.17	343.57	145.50			435.93	192.56	384.92	302.61	112.37	6.00	45.87	427.49	364.18	352.75	365.74	296.46	557.48		532.59	413.81	97.65	7.00	36.91
**29**	314.78	346.79	144.78			438.95	192.79	358.96	299.51	110.25	6.00	45.01	461.06	385.20	355.45	394.00	285.11	527.20		528.38	419.49	90.53	7.00	34.22
**30**	335.28	345.44	148.20			444.74	212.88	365.33	308.64	108.36	6.00	44.24	430.75	379.50	348.56	422.13	258.58	546.85		537.35	417.67	102.26	7.00	38.65

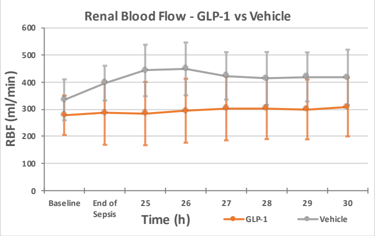

**Sheet**	Renal Vascular Conductance
**Graph**	GLP-1 vs Vehicle
**Title**	Renal Vascular Conductance - GLP-1 vs Vehicle
**Legend**	RVC (ml/min/mmHg)

	GLP-1												Vehicle											
**Time (h)**	**Gamgee**	**Took**	**Simba**	**Wiggins**	**Ratcliffe**	**Bashful**	**Sneezy**	**Shorty**	**MEAN**	**SD**	**N**	**SEM**	**Timon**	**Kocoum**	**Percy**	**Ben 2**	**Zazu**	**CPB101**	**Dumbo**	**Doc**	**MEAN**	**SD**	**N**	**SEM**
**Baseline**	2.87	2.17	2.84	4.24		4.11	2.66	3.82	3.24	0.80	7.00	0.30	3.95	3.05	3.28	3.79	3.39	4.84		3.99	3.76	0.60	7.00	0.23
**End of Sepsis**	4.35	4.18	1.05			5.89	3.25	4.71	3.91	1.64	6.00	0.67	5.62	4.22	4.40	4.97	4.18	5.99		5.48	4.98	0.74	7.00	0.28
**25**	4.62	3.68	1.28			5.65	2.77	4.65	3.77	1.56	6.00	0.64	6.00	5.06	4.66	4.36	4.14	6.61		8.56	5.63	1.57	7.00	0.59
**26**	4.03	4.43	1.30			6.26	2.96	5.04	4.00	1.72	6.00	0.70	6.36	5.24	4.61	4.83	4.49	7.72		8.63	5.99	1.64	7.00	0.62
**27**	4.46	4.68	1.52			5.59	3.16	5.22	4.11	1.51	6.00	0.62	6.27	5.95	4.04	3.98	4.27	7.09		7.69	5.61	1.53	7.00	0.58
**28**	4.51	5.01	1.75			5.30	2.91	5.31	4.13	1.47	6.00	0.60	6.54	4.80	4.49	4.40	4.09	6.84		7.86	5.58	1.48	7.00	0.56
**29**	4.19	4.99	1.73			4.46	2.75	5.08	3.87	1.34	6.00	0.55	6.68	5.68	4.37	4.38	3.86	6.68		7.98	5.66	1.53	7.00	0.58
**30**	4.49	4.67	1.79			5.34	3.14	5.07	4.08	1.36	6.00	0.55	6.15	5.34	4.06	5.38	3.21	6.74		7.60	5.50	1.51	7.00	0.57

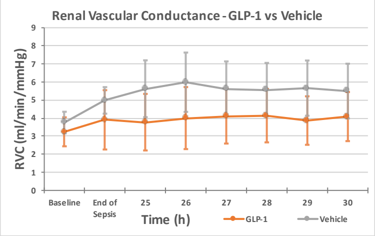

**Sheet**	Urine Output
**Graph**	GLP-1 vs Vehicle
**Title**	Urine Output - GLP-1 vs Vehicle
**Legend**	Urine Output (ml/h)

	GLP-1												Vehicle											
**Time (h)**	**Gamgee**	**Took**	**Simba**	**Wiggins**	**Ratcliffe**	**Bashful**	**Sneezy**	**Shorty**	**MEAN**	**SD**	**N**	**SEM**	**Timon**	**Kocoum**	**Percy**	**Ben 2**	**Zazu**	**CPB101**	**Dumbo**	**Doc**	**MEAN**	**SD**	**N**	**SEM**
**Baseline**	31.00	31.00	24.00	30.00	51.00	55.00	61.00	78.00	45.13	19.04	8.00	6.73	21.50	28.00	42.00	104.00	40.00	47.00	65.00	73.00	52.56	26.96	8.00	9.53
**End of Sepsis**	5.67	26.67	5.67	22.00	13.67	31.33	41.00	39.67	23.21	14.01	8.00	4.95	71.00	24.67	54.67	40.33	22.33	18.00	47.33	19.33	37.21	19.37	8.00	6.85
**25**	91.00	230.00	60.00	180.00	2.00	315.00	210.00	90.00	147.25	103.61	8.00	36.63	65.00	100.00	280.00	55.00	120.00	43.00	26.00	72.00	95.13	80.51	8.00	28.46
**26**	82.00	360.00	135.00	135.00	25.00	325.00	240.00	225.00	190.88	116.98	8.00	41.36	250.00	245.00	155.00	67.00	80.00	85.00	120.00	180.00	147.75	72.53	8.00	25.64
**27**	110.00	130.00	110.00	85.00	55.00	89.00	100.00	150.00	103.63	28.95	8.00	10.23	610.00	95.00	91.00	520.00	14.00	74.00	100.00	255.00	219.88	224.80	8.00	79.48
**28**	42.00	52.00	90.00	15.00	25.00	120.00	65.00	90.00	62.38	35.89	8.00	12.69	81.00	38.00	115.00	120.00	7.00	67.00	100.00	210.00	92.25	61.16	8.00	21.62
**29**	66.00	65.00	75.00	130.00	9.00	84.00	67.00	49.00	68.13	33.82	8.00	11.96	108.00	36.00	72.00	135.00	38.00	58.00	45.00	92.00	73.00	35.92	8.00	12.70
**30**	88.00	26.00	13.00	33.00	38.00	84.00	70.00	50.00	50.25	27.72	8.00	9.80	87.00	42.00	58.00	126.00	75.00	47.00	58.00	120.00	76.63	32.07	8.00	11.34

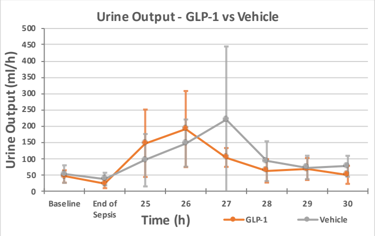

**Sheet**	Urinary Oxygenation
**Graph**	GLP-1 vs Vehicle
**Title**	Urinary Oxygenation - GLP-1 vs Vehicle
**Legend**	PuO2 (mmHg)

	GLP-1												Vehicle											
**Time (h)**	**Gamgee**	**Took**	**Simba**	**Wiggins**	**Ratcliffe**	**Bashful**	**Sneezy**	**Shorty**	**MEAN**	**SD**	**N**	**SEM**	**Timon**	**Kocoum**	**Percy**	**Ben 2**	**Zazu**	**CPB101**	**Dumbo**	**Doc**	**MEAN**	**SD**	**N**	**SEM**
**Baseline**	30.84	25.04				37.66		53.47	36.75	12.28	4.00	6.14	21.03	33.25	4.33		9.66	23.32		8.35	16.66	11.04	6.00	4.51
**End of Sepsis**	1.79	33.40				27.83		29.72	23.18	14.45	4.00	7.22	0.82	43.35	3.00		15.36	22.95		8.10	15.60	15.86	6.00	6.47
**25**	4.49	33.37				32.70		31.29	25.46	14.01	4.00	7.00	55.10	39.69	16.30		29.13	26.25		6.91	28.90	17.04	6.00	6.96
**26**	14.58	35.93				46.53		20.12	29.29	14.63	4.00	7.31	45.90	41.72	6.31		41.44	21.25		12.80	28.24	16.95	6.00	6.92
**27**	12.01	37.75				44.21		22.41	29.09	14.60	4.00	7.30	72.92	39.03	1.71		30.48	16.66		18.33	29.85	24.65	6.00	10.06
**28**	9.39	25.74				47.63		41.03	30.95	17.05	4.00	8.52	69.55	29.97	2.76		23.08	14.46		13.98	25.63	23.40	6.00	9.55
**29**	4.99	19.42				22.59		36.04	20.76	12.74	4.00	6.37	64.32	35.49	1.65		21.94	13.83		5.20	23.74	23.31	6.00	9.52
**30**	4.28	27.64				22.64		37.44	23.00	13.91	4.00	6.96	44.66	41.42	0.46		12.50	7.03		9.75	19.30	18.84	6.00	7.69

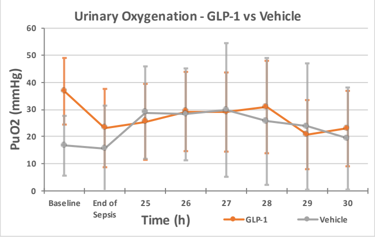

**Sheet**	Renal Cortical Perfusion
**Graph**	GLP-1 vs Vehicle
**Title**	Renal Cortical Perfusion - GLP-1 vs Vehicle
**Legend**	RCF (BPU)

	GLP-1												Vehicle											
**Time (h)**	**Gamgee**	**Took**	**Simba**	**Wiggins**	**Ratcliffe**	**Bashful**	**Sneezy**	**Shorty**	**MEAN**	**SD**	**N**	**SEM**	**Timon**	**Kocoum**	**Percy**	**Ben 2**	**Zazu**	**CPB101**	**Dumbo**	**Doc**	**MEAN**	**SD**	**N**	**SEM**
**Baseline**	953.08		1121.84	2553.13	2371.65	4007.94	2248.96	1688.14	2134.96	1030.76	7.00	389.59	2509.62	3037.39	2661.68	1457.87	1726.44	805.11		787.93	1855.15	903.00	7.00	341.30
**End of Sepsis**	838.21		197.39	712.28	247.07	2758.28	1495.21	3318.64	1366.73	1230.96	7.00	465.26	3704.27	2217.38	829.75	4485.33	2455.58	1630.95		2498.31	2545.94	1225.01	7.00	463.01
**25**	729.59		422.80	679.94	529.14	2082.21	1370.97	4856.89	1524.51	1580.56	7.00	597.40	2928.02	2480.09	1064.63	3175.44	1739.00	1493.47		2232.07	2158.96	769.19	7.00	290.73
**26**	666.52		599.00	797.06	392.01	2177.49	1233.94	4667.52	1504.79	1516.08	7.00	573.03	2198.17	2870.03	1156.51	3030.38	1972.81	1782.54		2972.04	2283.21	707.20	7.00	267.30
**27**	561.79		406.29	787.44	229.50	1238.64	1199.25	4277.78	1242.96	1391.08	7.00	525.78	2574.28	2740.72	981.87	4223.83	2004.83	1694.82		1028.74	2178.44	1130.94	7.00	427.45
**28**	601.62		316.59	622.67	150.71	1398.93	1249.95	4203.48	1220.56	1392.41	7.00	526.28	2636.98	2089.49	1129.79	4545.10	1933.41	1745.71		970.64	2150.16	1198.57	7.00	453.02
**29**	622.89		300.27	716.11	180.74	1207.57	1205.71	4454.60	1241.13	1471.75	7.00	556.27	2870.60	2235.37	1293.00	4594.26	1303.33	1463.99		837.03	2085.37	1298.56	7.00	490.81
**30**	587.18		251.00	654.16	173.27	1275.58	3634.92	4132.71	1529.83	1653.38	7.00	624.92	3161.75	1784.47	1204.48	3635.99	909.89	1464.76		949.49	1872.98	1093.23	7.00	413.20

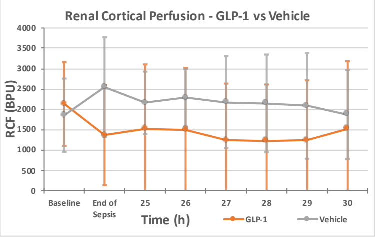

**Sheet**	Renal Cortical Oxygenation
**Graph**	GLP-1 vs Vehicle
**Title**	Renal Cortical Oxygenation - GLP-1 vs Vehicle
**Legend**	RCO2 (mmHg)

	GLP-1												Vehicle											
**Time (h)**	**Gamgee**	**Took**	**Simba**	**Wiggins**	**Ratcliffe**	**Bashful**	**Sneezy**	**Shorty**	**MEAN**	**SD**	**N**	**SEM**	**Timon**	**Kocoum**	**Percy**	**Ben 2**	**Zazu**	**CPB101**	**Dumbo**	**Doc**	**MEAN**	**SD**	**N**	**SEM**
**Baseline**	9.35	27.19		31.77	11.67	19.42	23.85	25.00	21.18	8.20	7.00	3.10	18.03	34.75		27.18	41.38			51.47	34.56	12.85	5.00	5.75
**End of Sepsis**	4.79	63.19		19.50	11.33	30.82	27.07	27.18	26.27	18.78	7.00	7.10	41.32	46.96		47.88	55.22			44.63	47.20	5.15	5.00	2.30
**25**	1.64	58.97		26.30	10.31	30.63	27.14	27.00	26.00	18.03	7.00	6.81	48.39	40.53		57.82	48.76			43.33	47.77	6.61	5.00	2.95
**26**	10.29	64.48		24.95	20.32	34.28	29.52	30.84	30.67	16.89	7.00	6.39	43.17	48.91		59.71	70.86			41.48	52.83	12.35	5.00	5.52
**27**	13.71	57.83		24.97	22.48	35.10	28.31	33.67	30.87	13.90	7.00	5.25	29.53	42.31		40.02	55.55			30.72	39.63	10.51	5.00	4.70
**28**	4.69	57.96		28.52	15.19	37.41	25.33	36.47	29.37	17.13	7.00	6.47	18.86	13.57		42.10	51.11			43.96	33.92	16.61	5.00	7.43
**29**	5.66	57.03		34.27	17.77	36.23	22.51	36.37	29.98	16.46	7.00	6.22	20.27	25.33		43.62	44.59			41.48	35.06	11.39	5.00	5.09
**30**	5.54	44.65		35.34	18.61	35.31	23.34	35.25	28.29	13.24	7.00	5.00	31.75	33.25		43.89	31.24			30.72	34.17	5.51	5.00	2.47

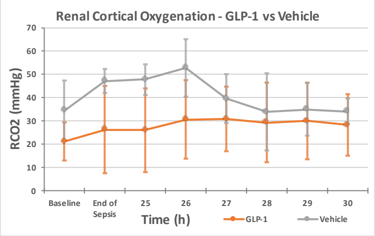

**Sheet**	Renal Medullary Perfusion
**Graph**	GLP-1 vs Vehicle
**Title**	Renal Medullary Perfusion - GLP-1 vs Vehicle
**Legend**	RMP (BPU)

	GLP-1												Vehicle											
**Time (h)**	**Gamgee**	**Took**	**Simba**	**Wiggins**	**Ratcliffe**	**Bashful**	**Sneezy**	**Shorty**	**MEAN**	**SD**	**N**	**SEM**	**Timon**	**Kocoum**	**Percy**	**Ben 2**	**Zazu**	**CPB101**	**Dumbo**	**Doc**	**MEAN**	**SD**	**N**	**SEM**
**Baseline**	517.19	1768.72	1416.02	657.85		2825.93	515.54	779.26	1211.50	858.15	7.00	324.35	1439.60	596.58	1163.87	837.87	493.10	662.86		524.39	816.90	358.53	7.00	135.51
**End of Sepsis**	837.17	433.39	113.20	128.68		1124.82	599.87	349.60	512.39	371.38	7.00	140.37	756.86	125.20	537.05	914.53	432.45	808.58		369.25	563.42	279.40	7.00	105.60
**25**	774.74	452.35	184.19	206.34		792.20	616.60	539.93	509.48	246.19	7.00	93.05	464.97	242.08	1083.03	749.64	519.89	832.34		1166.54	722.64	336.19	7.00	127.07
**26**	540.25	302.65	302.79	208.35		890.05	457.45	688.71	484.32	242.34	7.00	91.59	443.69	113.51	1159.92	182.00	557.98	828.08		878.58	594.82	383.09	7.00	144.79
**27**	735.94	392.70	290.49	215.90		1088.33	446.21	736.41	558.00	309.07	7.00	116.82	490.19	61.15	778.57	67.34	577.58	807.91		534.81	473.94	304.07	7.00	114.93
**28**	386.95	307.37	172.14	115.83		1179.47	340.16	748.65	464.37	375.31	7.00	141.85	636.00	56.54	685.92	35.10	467.57	796.18		636.82	473.45	307.79	7.00	116.33
**29**	295.65	279.50	101.48	165.08		1187.23	263.53	841.19	447.67	405.55	7.00	153.28	493.95	11.76	652.59	67.82	414.67	669.81		888.02	456.95	322.02	7.00	121.71
**30**	1149.10	485.13	40.53	239.72		1018.21	258.75	803.29	570.68	425.08	7.00	160.67	505.29	19.61	715.28	74.26	333.52	686.52		810.87	449.34	316.12	7.00	119.48

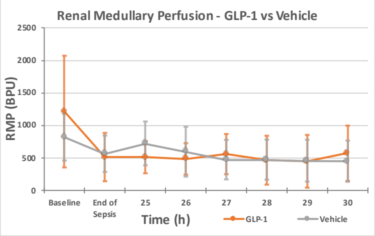

**Sheet**	Renal Medullary Oxygenation
**Graph**	GLP-1 vs Vehicle
**Title**	Renal Medullary Oxygenation - GLP-1 vs Vehicle
**Legend**	RMO2 (mmHg)

	GLP-1												Vehicle											
**Time (h)**	**Gamgee**	**Took**	**Simba**	**Wiggins**	**Ratcliffe**	**Bashful**	**Sneezy**	**Shorty**	**MEAN**	**SD**	**N**	**SEM**	**Timon**	**Kocoum**	**Percy**	**Ben 2**	**Zazu**	**CPB101**	**Dumbo**	**Doc**	**MEAN**	**SD**	**N**	**SEM**
**Baseline**	16.17	30.30	21.83		17.75	48.18	44.50	33.71	30.35	12.66	7.00	4.78	50.03	48.75	6.38	37.08	24.38	39.74		36.11	34.64	15.13	7.00	5.72
**End of Sepsis**	14.38	38.75	0.88		6.73	38.37	49.66	34.68	26.21	18.65	7.00	7.05	24.89	15.26	3.62		50.25	49.72		12.81	26.09	19.71	6.00	8.05
**25**	16.83	48.59	0.46		11.64	39.93	50.93	23.36	27.39	19.41	7.00	7.34	30.74	51.83	1.95		69.55			25.21	35.86	25.88	5.00	11.58
**26**	20.42	38.45	0.49		10.42	45.29	50.44	24.52	27.15	18.45	7.00	6.97	36.50	42.42	13.04		64.00			31.33	37.46	18.46	5.00	8.26
**27**	23.86	39.09	0.46		6.39	49.59	44.21	31.18	27.82	18.73	7.00	7.08	26.74	22.00	3.46		59.88			6.10	23.64	22.59	5.00	10.10
**28**	22.42	30.73	0.46		6.22	48.16	24.26	38.57	24.40	16.90	7.00	6.39	33.09	3.56	2.03		48.12			4.43	18.25	21.11	5.00	9.44
**29**	18.46	10.75	0.47		10.39	47.28	0.59	40.23	18.31	18.58	7.00	7.02	29.73	8.40	2.48		28.94			21.97	18.30	12.30	5.00	5.50
**30**	18.11	9.15	0.41		7.61	44.24	8.19	44.37	18.87	18.12	7.00	6.85	34.42	13.75	2.91		20.88			31.36	20.66	12.91	5.00	5.77

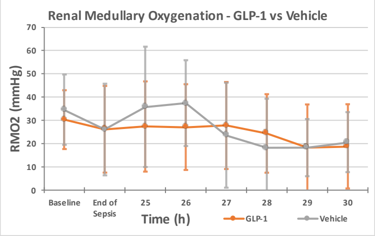

**Sheet**	Creatinine
**Graph**	GLP-1 vs Vehicle
**Title**	Creatinine - GLP-1 vs Vehicle
**Legend**	Creat (mcmol/l)

	GLP-1												Vehicle											
**Time (h)**	**Gamgee**	**Took**	**Simba**	**Wiggins**	**Ratcliffe**	**Bashful**	**Sneezy**	**Shorty**	**MEAN**	**SD**	**N**	**SEM**	**Timon**	**Kocoum**	**Percy**	**Ben 2**	**Zazu**	**CPB101**	**Dumbo**	**Doc**	**MEAN**	**SD**	**N**	**SEM**
**Baseline**	70.00	66.00	63.00	44.00	83.00	62.00	74.00	62.00	65.50	11.29	8.00	3.99	62.00	64.00	48.00	62.00	80.00	64.00	63.00	66.00	63.63	8.65	8.00	3.06
**End of Sepsis**	113.00	79.00	156.00	114.00	163.00	83.00	88.00	78.00	109.25	34.11	8.00	12.06	89.00	104.00	75.00	97.00	99.00	104.00	131.00	112.00	101.38	16.38	8.00	5.79
**26**	98.00	70.00	141.00	103.00	152.00	69.00	81.00	69.00	97.88	32.83	8.00	11.61	82.00	89.00	70.00	78.00	90.00	81.00	126.00	99.00	89.38	17.17	8.00	6.07
**28**	87.00	73.00	131.00	97.00	131.00	65.00	77.00	63.00	90.50	27.33	8.00	9.66	82.00	86.00	71.00	72.00	85.00	79.00	118.00	102.00	86.88	15.86	8.00	5.61
**30**	85.00	72.00	120.00	92.00	121.00	62.00	80.00	57.00	86.13	24.10	8.00	8.52	84.00	84.00	71.00	73.00	86.00	76.00	115.00	91.00	85.00	13.94	8.00	4.93

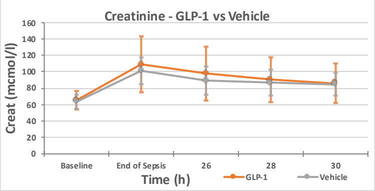

**Sheet**	Creatinine Clearance
**Graph**	GLP-1 vs Vehicle
**Title**	Creatinine Clearance - GLP-1 vs Vehicle
**Legend**	Creat Clear (ml/min)

	GLP-1												Vehicle											
**Time (h)**	**Gamgee**	**Took**	**Simba**	**Wiggins**	**Ratcliffe**	**Bashful**	**Sneezy**	**Shorty**	**MEAN**	**SD**	**N**	**SEM**	**Timon**	**Kocoum**	**Percy**	**Ben 2**	**Zazu**	**CPB101**	**Dumbo**	**Doc**	**MEAN**	**SD**	**N**	**SEM**
**Baseline**	78.98	20.35	41.90	40.91	49.16	53.23	153.87	50.32	61.09	40.85	8.00	14.44	74.56	29.17	94.79	103.44	63.33	85.68	84.26	101.39	79.58	24.40	8.00	8.63
**End of Sepsis**	9.97	27.66	4.33	7.86	20.22	40.46	57.61	41.73	26.23	19.08	8.00	6.74	54.61	70.51	28.80	54.19	50.00	47.96	35.99	65.04	50.89	13.78	8.00	4.87
**26**	80.88	240.00	23.94	80.83	28.51	329.71	167.90	152.17	137.99	106.76	8.00	37.74	91.46	188.11	166.07	52.97	133.33	64.71	49.21	57.58	100.43	54.96	8.00	19.43
**28**	87.70	59.36	19.47	10.05	51.21	104.62	56.28	109.52	62.28	36.64	8.00	12.95	59.27	84.69	126.88	102.78	10.57	91.88	110.17	85.78	84.00	35.75	8.00	12.64
**30**	138.04	58.38	9.75	52.01	88.98	45.16	128.33	78.95	74.95	43.06	8.00	15.23	93.21	24.17	130.70	115.07	55.23	61.84	52.12	112.09	80.55	37.49	8.00	13.26

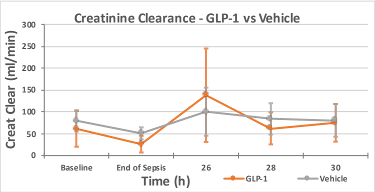

**Sheet**	Fractional Excretion Sodium
**Graph**	GLP-1 vs Vehicle
**Title**	Fractional Excretion Sodium - GLP-1 vs Vehicle
**Legend**	FE Na (%)

	GLP-1												Vehicle											
**Time (h)**	**Gamgee**	**Took**	**Simba**	**Wiggins**	**Ratcliffe**	**Bashful**	**Sneezy**	**Shorty**	**MEAN**	**SD**	**N**	**SEM**	**Timon**	**Kocoum**	**Percy**	**Ben 2**	**Zazu**	**CPB101**	**Dumbo**	**Doc**	**MEAN**	**SD**	**N**	**SEM**
**Baseline**	1.15	1.11	0.79	0.33	2.44	1.16	0.62	0.84	1.06	0.63	8.00	0.22	1.18	1.29	0.67	0.62	0.86	1.02	1.10	2.88	1.20	0.72	8.00	0.25
**End of Sepsis**	0.40	1.42	7.63	0.88	0.90	0.29	0.85	0.11	1.56	2.49	8.00	0.88	1.62	0.28	0.98	0.21	1.01	0.13	0.45	0.17	0.61	0.54	8.00	0.19
**26**	1.91	2.95	7.35	2.48	0.81	1.27	1.93	0.34	2.38	2.18	8.00	0.77	2.24	1.26	2.11	0.43	1.37	0.43	0.93	0.52	1.16	0.72	8.00	0.26
**28**	1.42	1.57	4.02	2.83	0.54	1.39	0.68	0.34	1.60	1.26	8.00	0.44	3.22	0.50	2.00	0.66	1.99	0.42	1.13	1.50	1.43	0.96	8.00	0.34
**30**	1.64	1.34	1.59	1.12	0.64		0.57	0.50	1.06	0.49	7.00	0.18	1.96	0.82	0.86	0.59	2.41	0.24	2.05	2.08	1.38	0.83	8.00	0.29

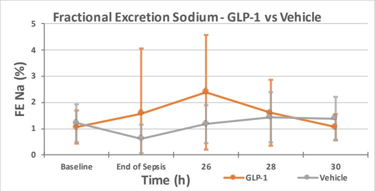

**Sheet**	Renal Oxygen Delivery
**Graph**	GLP-1 vs Vehicle
**Title**	Renal Oxygen Delivery - GLP-1 vs Vehicle
**Legend**	Renal DO2 (ml/min)

	GLP-1												Vehicle											
**Time (h)**	**Gamgee**	**Took**	**Simba**	**Wiggins**	**Ratcliffe**	**Bashful**	**Sneezy**	**Shorty**	**MEAN**	**SD**	**N**	**SEM**	**Timon**	**Kocoum**	**Percy**	**Ben 2**	**Zazu**	**CPB101**	**Dumbo**	**Doc**	**MEAN**	**SD**	**N**	**SEM**
**Baseline**	36.69	17.69	31.42	48.17		48.01	28.66	45.95	36.65	11.54	7.00	4.36	37.69	30.94	29.60	44.12	33.23	48.85		54.06	39.78	9.43	7.00	3.57
**End of Sepsis**	36.65	30.80	11.16			53.47	28.56	45.85	34.41	14.75	6.00	6.02	45.61	48.26	42.15	54.56	41.59	64.94		65.76	51.84	10.19	7.00	3.85
**26**	27.71	31.30	10.67			50.47	28.59	43.91	32.11	13.92	6.00	5.68	44.66	54.09	37.35	51.89	38.52	75.81		59.96	51.75	13.44	7.00	5.08
**28**	34.65	32.81	15.77			53.11	25.17	46.73	34.71	13.69	6.00	5.59	44.56	46.45	34.34	41.83	33.34	68.08		59.61	46.89	12.80	7.00	4.84
**30**	36.88	32.53	15.91			52.70	27.37	46.25	35.27	13.20	6.00	5.39	42.45	49.49	36.38	49.51	31.73	62.88		61.30	47.68	11.78	7.00	4.45

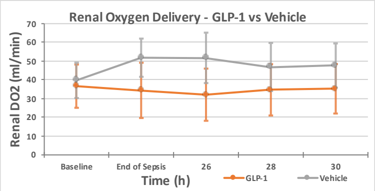

**Sheet**	Renal Oxygen Consumption
**Graph**	GLP-1 vs Vehicle
**Title**	Renal Oxygen Consumption - GLP-1 vs Vehicle
**Legend**	Renal VO2 (ml/min)

	GLP-1												Vehicle											
**Time (h)**	**Gamgee**	**Took**	**Simba**	**Wiggins**	**Ratcliffe**	**Bashful**	**Sneezy**	**Shorty**	**MEAN**	**SD**	**N**	**SEM**	**Timon**	**Kocoum**	**Percy**	**Ben 2**	**Zazu**	**CPB101**	**Dumbo**	**Doc**	**MEAN**	**SD**	**N**	**SEM**
**Baseline**		2.94	5.02			7.01		4.98	4.99	1.66	4.00	0.83	3.63	3.00	5.90	5.95				7.59	5.21	1.88	5.00	0.84
**End of Sepsis**		2.24				4.89		5.20	4.11	1.63	3.00	0.94	3.83	4.84	4.55	4.55				5.26	4.61	0.52	5.00	0.23
**26**		4.17				6.03		3.97	4.72	1.14	3.00	0.66	4.86	5.79	5.32	10.51				3.70	6.04	2.62	5.00	1.17
**28**		3.23				5.97		5.82	5.00	1.54	3.00	0.89	3.85	5.02	3.46	9.08				5.15	5.31	2.23	5.00	1.00
**30**		3.30				5.35		6.78	5.14	1.75	3.00	1.01	1.33	4.16	6.93	8.42				5.23	5.21	2.71	5.00	1.21

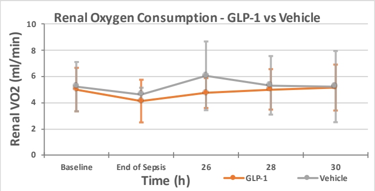

**Sheet**	Glycaemia
**Graph**	GLP-1 vs Vehicle
**Title**	Glycaemia - GLP-1 vs Vehicle
**Legend**	Plasma Glucose (mmol/l)

	GLP-1												Vehicle											
**Time (h)**	**Gamgee**	**Took**	**Simba**	**Wiggins**	**Ratcliffe**	**Bashful**	**Sneezy**	**Shorty**	**MEAN**	**SD**	**N**	**SEM**	**Timon**	**Kocoum**	**Percy**	**Ben 2**	**Zazu**	**CPB101**	**Dumbo**	**Doc**	**MEAN**	**SD**	**N**	**SEM**
**Baseline**	2.80	2.90	2.50	2.10	3.10	2.90	2.60	2.80	2.71	0.31	8.00	0.11	3.40	3.20	2.80	2.90	3.60	3.90	2.54	3.30	3.21	0.44	8.00	0.16
**End of Sepsis**	1.70	3.10	2.30	3.30	3.50	4.20	3.20	1.70	2.88	0.89	8.00	0.32	1.90	1.20	1.70	2.40	2.40	7.60	4.10	1.60	2.86	2.11	8.00	0.74
**26**	2.40	3.30	4.10	3.10	1.30	4.20	2.30	2.30	2.88	0.99	8.00	0.35	2.40	1.70	2.70	6.80	2.70	2.20	5.30	1.80	3.20	1.84	8.00	0.65
**28**	2.30	2.60	2.70	4.10	2.80	3.70	1.70	2.50	2.80	0.77	8.00	0.27	1.80	1.40	5.30	4.60	2.10	5.20	6.90	1.70	3.63	2.11	8.00	0.75
**30**	2.40	3.30	2.40	3.30	3.00	4.30	1.70	2.60	2.88	0.79	8.00	0.28	2.40	1.80	6.60	2.70	2.60	5.50	6.20	1.40	3.65	2.09	8.00	0.74

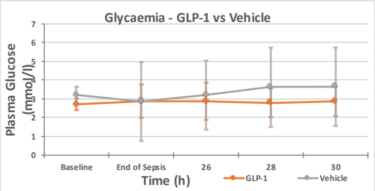

Supplement: Supplementary file 1 — Supplementary Material 1 [file 41598_2025_33109_MOESM1_ESM.pdf]
