# Supplementary material for: Effects of glucagon-like peptide-1 on systemic hemodynamics, kidney function, and intrarenal oxygenation in sheep with sepsis-associated acute kidney injury
Source: Sci Rep. 2025 Dec 24;16:3250. doi: 10.1038/s41598-025-33109-0 (PMC12830701; doi:10.1038/s41598-025-33109-0)
Supplement: Supplementary file 2 — Supplementary Material 2 [file 41598_2025_33109_MOESM2_ESM.pdf]

Supplementary Data S2 (Table). Number of sheep (out of 8) that satisfied the criteria for each of the histopathological abnormalities observed in each treatment group.

Scoring criteria	**Acute tubular necrosis**	**Inflammatory cells present**	**Interstitial fibrosis**	**Tubular casts**	**Red blood cells**
**GLP-1**					
- negative	4	4	8	5	4
+/- focal	1	1	0	2	0
++ diffuse	3	3	0	1	4
**Vehicle**					
- negative	6	4	8	5	6
+/- focal	1	3	0	2	1
++ diffuse	1	1	0	1	1

Sections of the renal cortex and medulla were collected at autopsy following 48 h of recovery in sheep that received either GLP-1 (n=8) or vehicle treatment (n=8) during gram-negative sepsis. Formalin-fixed paraffin-embedded renal tissue was subjected to histochemical and immunohistochemical analysis for acute tubular necrosis (focal or diffuse denudation and/or flattening of tubular epithelial cells with tubular denudation, interstitial edema, loss of proximal tubular brush borders and tubular casts), interstitial mononuclear infiltrates (peritubular), fibrosis (interstitial), casts (hyaline, cellular or granular) and birefringence (oxalate) crystals. The grading system used included Grade 1 (0) = no change compared with the morphology of kidneys collected from healthy sheep; Grade 2 (+/-) = focal change in 5–30% of the total section; Grade 3 (++) = focal change in 30–50% of the total section.
